# Bibliometric Analysis of the 100 Most-Cited Clinical Trials on Gingival Recession Treatment: Trends in Flap Design, Biomaterials, and Global Contributions

**DOI:** 10.3390/jfb16100364

**Published:** 2025-10-01

**Authors:** Bartłomiej Górski, Kacper Nijakowski, Ilham Mounssif, Martina Stefanini, Anna Skurska

**Affiliations:** 1 Department of Periodontal and Oral Mucosa Diseases, Medical University of Warsaw, 02-097 Warsaw, Poland; bartlomiej.gorski@wum.edu.pl; 2 Department of Conservative Dentistry and Endodontics, Poznan University of Medical Sciences, 60-812 Poznan, Poland; kacpernijakowski@ump.edu.pl; 3 Department of Biomedical and Neuromotor Sciences, Bologna University, 40126 Bologna, Italy; ilham.mounssif2@unibo.it (I.M.); martina.stefanini2@unibo.it (M.S.); 4 Department of Integrated Dentistry, Medical University of Bialystok, 15-276 Bialystok, Poland

**Keywords:** enamel matrix derivative, gingival recession, guided tissue regeneration, mucogingival defect, periodontal plastic surgery, soft tissue graft, periodontitis, periodontal diseases

## Abstract

Background: The aim of this bibliometric study was to evaluate publication trends in the most frequently cited clinical trials on the treatment of gingival recession, taking into account the augmentation materials used. Methods: A Web of Science search was performed among articles published by 30 September 2024. Two independent reviewers evaluated year of publication, journal, authorship country of authors, collaborative relationship, keywords, and the main domains. Results: The top one hundred most-cited clinical trials were published in the span of 26 years from 1993 to 2019, and the total citation counts varied from 44 to 284 (83.69 citations per paper). There was correlation between the time of publication and the number of citations. The articles were authored by 333 researchers representing twenty-two countries. Italy contributed the highest number of articles (*n* = 36), followed by the USA (*n* = 28) and Brazil (*n* = 17). International collaborations were predominantly observed between Italy, the USA, and Switzerland. The type of graft was the most cited field of research (34), followed by guided tissue regeneration (17) and enamel matrix derivative (13). Conclusions: The country that produced the highest number publications among the 100 most-cited clinical trials on gingival recession treatment was Italy. The use of connective tissue graft (CTG) and coronally advanced flap (CAF) was the most prominent trend. Future work should combine bibliometric mapping with critical quality appraisal and explore whether citation trends align with best available evidence.

## 1. Introduction

Gingival recession (GR) is described as the exposure of the root surface following the apical migration of the gingival margin (GM) in relation to the cementoenamel junction (CEJ) [1]. GR affects both younger and older populations with a prevalence of up to 70% in individuals aged >50 years [2]. This condition may be caused by several factors, such as improper tooth brushing, plaque-induced inflammation, periodontal disease, intrasulcular restorative or prosthetic cervical margin placement, and orthodontic treatment [3].

GRs are classified based on interproximal clinical attachment loss into the following: recession type 1 (RT1) with no loss of interdental attachment, recession type 2 (RT2) when inter-dental attachment loss is smaller than buccal attachment loss, and recession type 3 (RT3) when interdental attachment loss is greater than buccal attachment loss [4]. This system overcomes some drawbacks of the previously used Miller classification [5]. GRs may be associated with the excessive length of teeth, an impaired esthetic appearance, improper plaque control, caries or non-caries cervical lesions and teeth hypersensitivity [6]. Consequently, GRs can affect the perceived oral health-related quality of life.

Several surgical techniques were introduced for the treatment of GRs. The coronally advanced flap (CAF) with autogenous connective tissue graft (CTG) was coined the “gold standard” [7]. A Cochrane systematic review and meta-analysis indicated that CAF alone, CAF with subepithelial connective tissue graft (SCTG), or CAF together with different biomaterials may be considered the flap of choice for GR treatment [8]. In cases where both root coverage and gain in the width of keratinized tissue (KTW) were required, the use of CTG enhanced clinical outcomes. Moreover, CAF demonstrated superior results to tunnel technique (TUN) when the same graft was used in both techniques [9].

There has been a continuous development in research publications focusing on GRs in terms of treatment modalities, materials, and growth factors. Nevertheless, the literature lacks bibliometric quantitative and qualitative analysis. Citation analyses are used increasingly to evaluate scientific production in case of individual scientists, rankings of universities and research institutions or just for the evaluation of the importance of publications [10]. This tool can provide useful insights into contemporary trends and emerging collaborations between different scientific teams [11]. As a result, bibliometric analysis may guide researchers toward further cooperation or focus on areas where exploration activity is required [12]. A highly cited paper might impact both research and clinical practice. Randomized controlled trials (RCTs) are widely recognized as having the highest level of evidence and help guide clinicians in their decision-making to maximize patient outcomes. However, RCTs require high-level expertise, optimal resources, and substantial time investment. Therefore, the aim of this study was to evaluate publishing and citation trends in the top one hundred most-cited clinical trials on GRs treatment, considering the augmentation materials used.

## 2. Materials and Methods

### 2.1. Study Design

This bibliometric analysis was designed in line with the appropriate methodological guidelines [13]. As it was a retrospective evaluation of publicly available data no Institutional Review Board approval and trial registration was required (Clinical trial number: not applicable).

### 2.2. Bibliometric Search Strategy and Data Extraction

The search was conducted using the Web of Science database belonging to Clarivate. The following search formula was used: TS = ((“gingival recession” OR recession OR “recession defect” OR “mucogingival defect” OR “denuded root” OR “exposed root” OR “peri-implant deficiencies”) AND (surgery OR “plastic surgery” OR “periodontal plastic surgery” OR “periodontal surgery” OR regeneration OR “guided tissue regeneration” OR “soft tissue regeneration” OR “soft tissue graft” OR “soft tissue grafting” OR “tissue transplantation” OR “acellular dermis” OR “collagen matrix” OR “xenogenic matrix graft” OR “enamel matrix protein” OR “hyaluronic acid” OR “biological product” OR “platelet-rich fibrin” OR “platelet-rich plasma” OR “coronally advanced flap” OR “laterally positioned flap” OR “tunnel technique”)).

**Figure 1:**
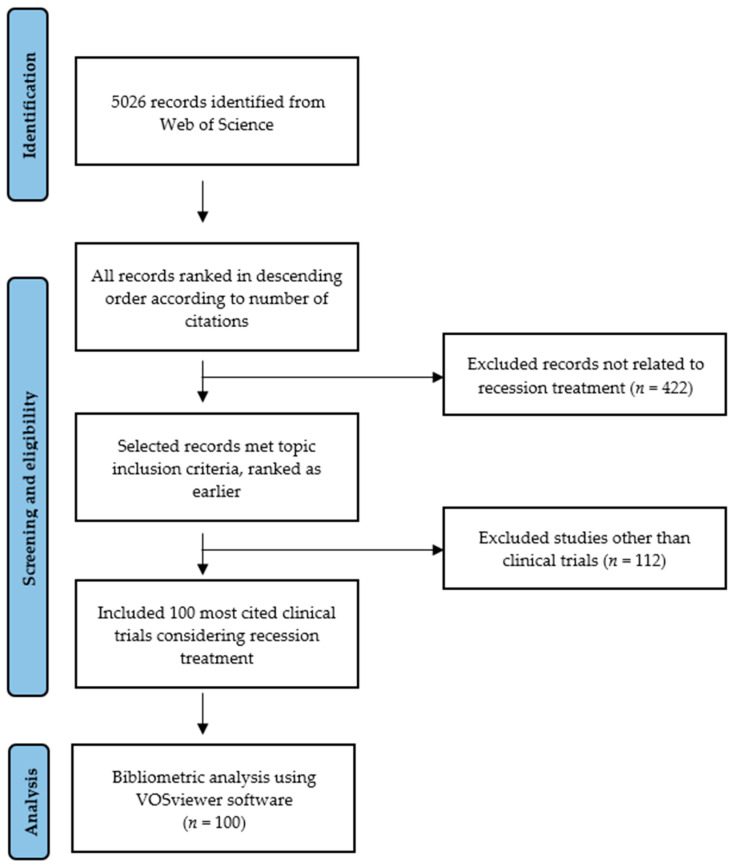
Flow chart presenting the search process.

By 30 September 2024, 5026 records were found in the database. The searched works were not limited by publication date; only a filter for the English language and the publication type “article” was applied. Among which the 100 most cited English-language clinical trials focusing on gingival recession treatment were selected manually (Table S1: List of 100 most-cited clinical trials on gingival recession treatment with total citations). Only RCT (randomized controlled trials) were included for the analysis. The exclusion criteria were publications defined as: expert opinions, animal and cell studies, case reports, case-series, case–control studies, cohort studies, systematic reviews. Two researchers (B.G. and A.S.) made the selection based on titles, abstracts, and full texts. Disagreements between the investigators were resolved through discussion. The search process is shown in Figure 1. The main domain of the articles was identified. The data concerning the included studies were exported into an Excel spreadsheet directly from WoS.

### 2.3. Statistical Analysis

Data on selected records were taken directly from the Web of Science database as a text file and imported for analysis in VOSviewer 1.6.20 (a bibliometric software program from the Centre for Science and Technology Studies of Leiden University). The evaluated bibliometric parameters included the number of citations, the year of publication, authorship, country of authors, institution of publications, international research collaborations, keywords, published journals, and research domains. Based on bibliographic data, citation and keyword co-occurrence maps were created. In the map, the bubble size demonstrated the number of publications, while the distance between them their relatedness. In the network visualization, the same-colored bubbles formed clusters, indicating close collaboration. Detailed information is available in the figure legends. Spearman correlation analysis was performed using Statistica 13.3 (StatSoft, Cracow, Poland). The level of statistical significance was set at α = 0.05.

## 3. Results

**Figure 2:**
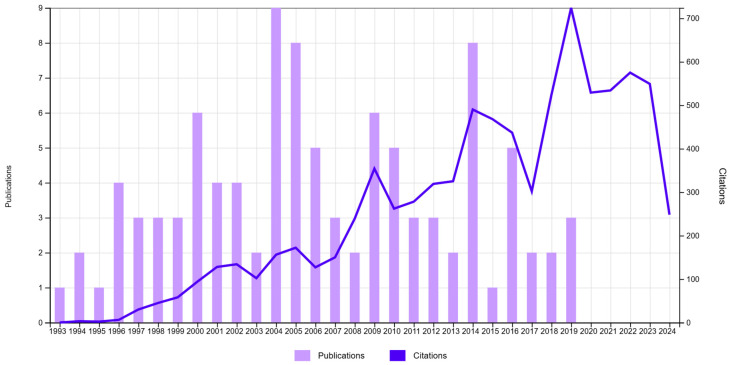
Histogram showing the publication dates of the selected 100 most cited clinical trials with the number of their citations (see Table S1 for full records of the top 100 articles).

**Figure 3:**
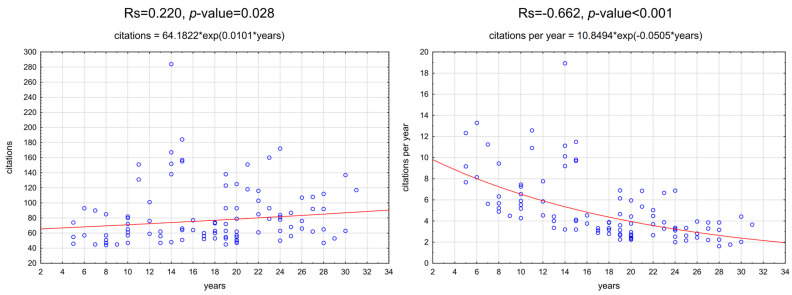
Spearman correlations between the number of citations (**left**)/citation density (**right**) and age of publication with exponential fitting.

The 100 most-cited clinical trials on GRs treatment were authored by 333 authors from 103 organizations. Those organizations represented 22 countries. The time intervals of the included publications with their citations are presented in the histogram (Figure 2). All included articles were published in English. They were published in the span of 26 years from 1993 to 2019. The average age of publications was 17.64 ± 6.74 years. The highest number of most-cited articles (*n* = 9) were published in the year 2004, followed by 2005/2014 and 2000/2009 with eight and six articles, respectively. Significant correlations were found between the number of citations or citation density and the age of publication (Figure 3). The highest citation impact (157.8 citation/article) was gained from five articles published in 2010, and the lowest citation impact (45 citation/article) was received from one article published in 2015. Seventy one percent of the articles were published in the first 18 years (1993–2010), while 29% of the articles were published from 2011 onward.

The total citation count varied from 44 to 284 with 83.69 citations per paper on average. The highest cited paper was “Patient morbidity and root coverage outcome after subepithelial connective tissue and de-epithelialized grafts: a comparative randomized-controlled clinical trial” by Zucchelli et al. [14] published in the *Journal of Clinical Periodontology* and was cited 284 times. The top 25 most-cited articles are presented in Table 1.

**Figure 4:**
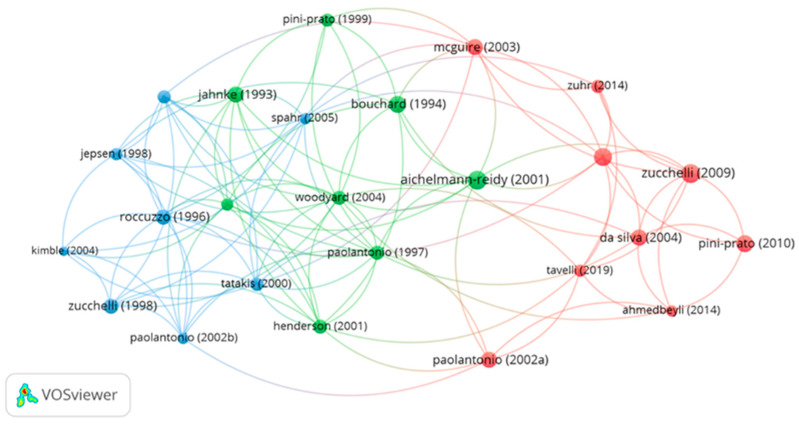
The network visualization for 25 publications with the most citation links based on citation analysis (the same-colored bubbles formed clusters, indicating close collaboration).

Figure 4 shows the visualization of the citation network for 25 articles with the largest number of connections. Among them, the most links were demonstrated by Woodyard et al. (2004) [Table S1] located in the central green cluster connecting two adjacent clusters–the blue one with more connections and the red one with fewer and weaker connections. Three main color groups indicate close cooperation between the authors of the same collection.

A total of 333 writers were identified, and 231 (69.4%) contributed to a single article. Twelve authors had more than five publications on the list. In the top five of the most productive and most cited authors, we found the same names, only in different order. In both rankings, Cortellini leads with 12 articles and 1112 citations (average 92.7 citations per paper) ahead of Zucchelli with 10 articles and 1174 citations (average 117.4 citations per paper). However, the highest citation rate of these five was presented by De Sanctis (146.8–6 articles with 881 citations). Detailed data are presented in Table 2. An average of five authors contributed to the 100 most-cited articles, and only two articles were found with a single author. The six author pattern was found more frequently with twenty-three articles.

**Figure 5:**
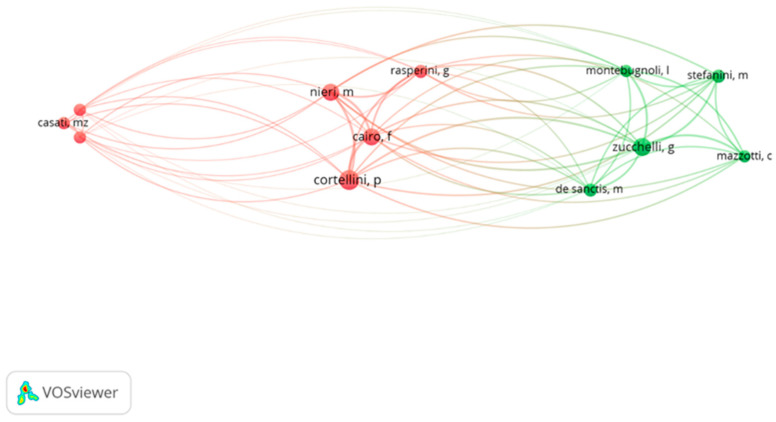
The network visualization for authors with a minimum of five publications based on citation analysis (the same-colored bubbles formed clusters, indicating close collaboration).

Figure 5 presents a network of links based on mutual citations between twelve authors who had at least five in the top 100 papers. The highest total link strength was characterized by Pierpaolo Cortellini (179, according to VOSviewer calculations).

Most papers were affiliated with the University of Bologna and the University of Florence—10 publications each, which were cited as many as 1174 times (index of 117.4 citations per record), and 996 times (index of 99.6 citations per record), respectively. The University of Siena had the highest rate of cited papers—127.6 (1021 citations per eight published papers). Table 3 shows the top 10 organizations with the highest number of papers and citations.

**Figure 6:**
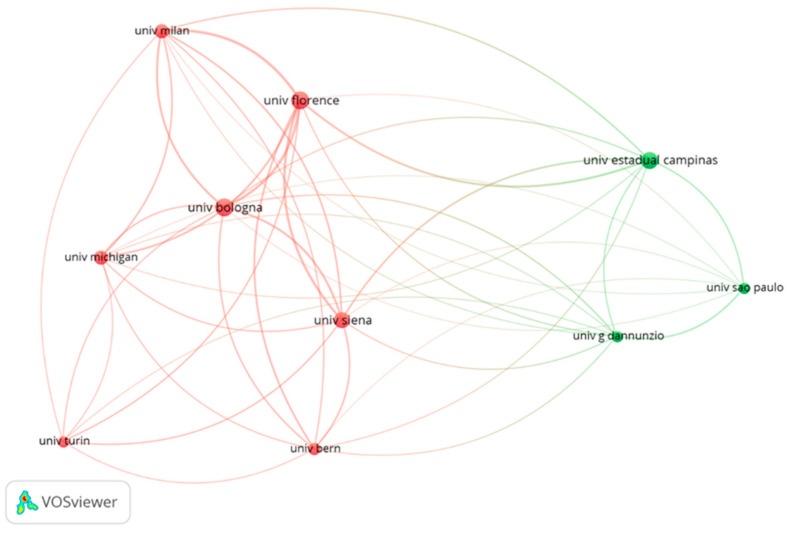
The network visualization for top 10 of universities based on citation analysis (the same-colored bubbles formed clusters, indicating close collaboration).

In the network of university links, the Italian centers dominated (Figure 6). The University of Florence had the highest total link strength (110), although it did not have the highest number of citations. In addition, two Brazilian universities, one American and one Swiss university were also identified. Two main color groups indicate close cooperation between the authors of the same collection.

Considering the nationality of the publications, the decisive leader was Italy–36 papers with 3386 citations (index 94.1), followed by the USA (28 papers and 3386 citations) and Brazil (17 papers and 1087 citations). Hungary had the highest citation rate–162.3 (3 papers with 487 citations). In the top 10 there were as many as 8 countries from Europe (Table 4).

**Figure 7:**
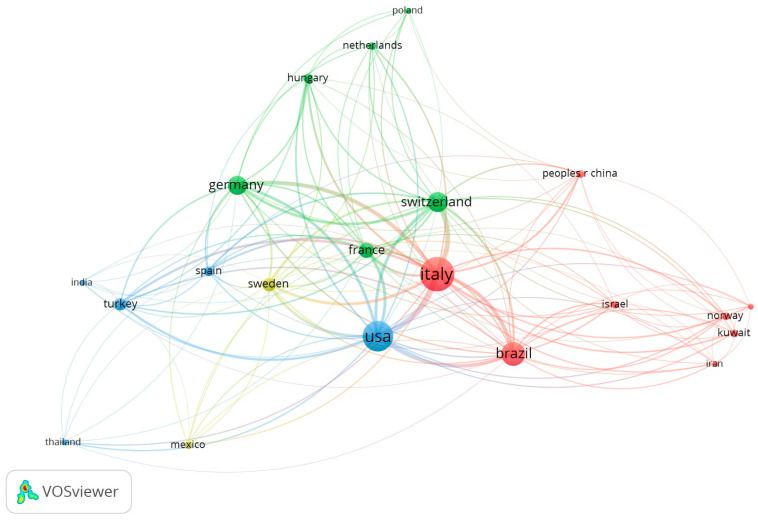
Network visualization for all countries based on citation analysis (the same-colored bubbles formed clusters, indicating close collaboration).

The analysis of links between countries considered all, even those with one published paper in the top 100 (Figure 7). In the presented network the clear dominance of Italy was observed (total link strength 564), ahead of the USA (total link strength 430) and Switzerland (total link strength 287).

Most of the articles were published in the *Journal of Periodontology* (59 papers with 4939 citations), and a considerable proportion in the *Journal of Clinical Periodontology* (37 papers with 3224 citations). The remaining four papers were published in three other journals (Table 5).

**Figure 8:**
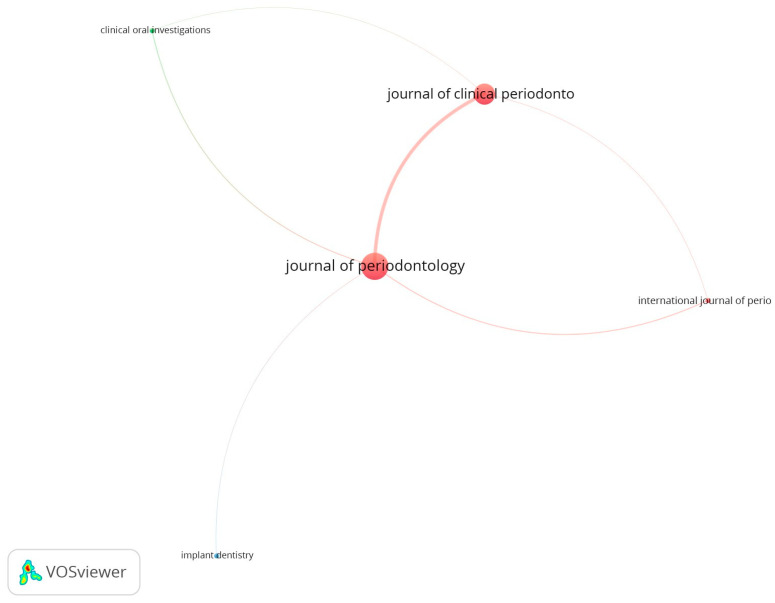
The network visualization for all journals is based on the citation analysis (the same-colored bubbles formed clusters, indicating close collaboration).

The network of links between journals was mainly based on the axis between two of them: *Journal of Periodontology* (total link strength 235) and *Journal of Clinical Periodontology* (total link strength 211)—Figure 8. Two main bubbles indicate the most influential journals.

Four hundred and twenty-four keywords were indexed in analyzed 100 papers. Table 6 depicts the most common twenty keywords. The term “root coverage” appeared most often—60 times, ahead of “gingival recession” occurring 42 times.

**Figure 9:**
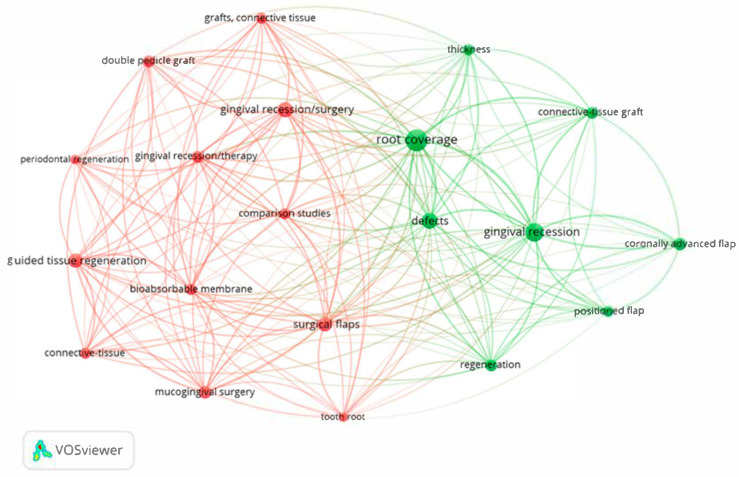
The network visualization for the top 20 keywords based on co-occurrence analysis (the same-colored bubbles formed clusters, indicating close collaboration).

The graphical links for the co-occurrence analysis for these keywords are presented in Figure 9. The largest total link strength presented the keyword “root coverage” (233, representative of the green cluster), followed by the keyword “gingival recession/surgery” (153, representative of the red cluster). Each cluster (green and red) groups keywords that tend to appear together in research, showing thematic subfields.

Table 7 summarizes the main research domains from the included studies considering the flap design and applied biomaterials (if used). Thirty-four articles concentrated on the type of graft (34%), 17 articles (17%) on GTR while 13 manuscripts (13%) concerned EMD application in the recession treatment. Fifty-nine studies were dedicated to assessing CTG, eighteen focused on ADMG, whereas eight analyzed XCM. The analysis also revealed that 84 articles focused on CAF, while only eight evaluated different flap designs (three articles assessed TUN technique, three articles double pedicle flap, and two repositioned flaps). Moreover, eight papers compared CAF with other surgical approaches (six manuscripts with TUN, one manuscript with semilunar coronally positioned flap, and one manuscript with modified envelope technique).

## 4. Discussion

This study thoroughly evaluated the bibliometric indicators of the one hundred most-cited randomized clinical trials on gingival recession treatment as the first study of its kind. The evaluated articles were ranked based on their total citation counts, which represented their cumulative impact. The key domains of the one hundred articles were also identified. We also examined how the process of scientific collaboration works between different centers. Presented findings may enhance better understanding of research trends in terms of international collaboration, fields of study, and treatment modalities. Furthermore, it can be assumed that a high number of citations may reflect the merit of the study and the prestige of the journal.

Articles with more than 100 citations can be regarded as highly influential in both research and clinical practice. Our findings revealed a significant association between the age of the articles and the number of citations. Nearly seventy percent of the clinical trials were published in the first 18 years, before 2010. It was the period of the most dynamic development of research in this field. However, the need for continuous improvement of scientific standards led to significant changes in legal regulations that occurred over time and, to some extent, set limitations on conducting research. This undoubtedly translated into their number. It can be assumed that older manuscripts tend to accumulate more citation counts due to their longer period of exposure. Interestingly, the articles published from 2000 to 2014 had the highest citation impact. The highest citation impact was achieved by five articles which were published in 2010. Authors of another bibliometric study [39] reported similar tendencies. More than half of the most-cited papers were published in two periodontal journals: *Journal of Periodontology* and *Journal of Clinical Periodontology*. Consequently, they are the most cited journals in the field of periodontics surgery [40]. The prominent position of both journals has been consistently observed. Ahmed and Slots stated that the *Journal of Clinical Periodontology*, *Journal of Periodontology*, and *Periodontology 2000* are three core periodontal journals [41]. These journals were among the 10 most-cited dental journals in the 2015–2019 period and carry on publishing high-impact articles. Similarly, Clinical Oral Implants Research followed by *Journal of Clinical Periodontology* and *Journal of Periodontology*, were the most cited journals on the topic of peri-implantitis [40].

Italy contributed the highest number of the most-cited manuscripts GR treatment, followed by the USA and Brazil. Italy, the USA, and Switzerland were the top-ranked countries in international research collaboration. The authors from these countries contributed 81% of the articles and accrued more than six thousand citations. However, papers produced by Hungary received the highest citation rate. The University of Bologna and the University of Florence affiliated most papers. Given the circumstances, Italy was the most productive country in terms of most-cited clinical research on GR treatment. Other studies endorsed that the USA, followed by Japan, were the most productive countries in the field of periodontal regeneration [39,42]. Another bibliometric analysis concluded that research published by Australia and Sweden received a higher ratio of citations [43]. Among the 100 most-cited articles on peri-implantitis, the top three locations of science production were Sweden (*n* = 31), Germany (*n* = 15), and Switzerland (*n* = 13) [40]. The present bibliometric study contributes to a more comprehensive understanding of the global landscape of research in GR treatment. The noticeable dominance of certain scientific centers indicates the pressing need for further research development and scientific collaboration between different universities and research institutions within their regional context, and around the world. It may be conducive to exploring new research trends, identifying emerging themes, promoting partnership, and preventing research redundancy.

Our analysis identified that a total of 333 writers contributed to the most-cited clinical trials on GRs. Among those, almost 70% contributed to a single paper. The average number of authors per paper was five and only two articles were found with a single author. Quite similarly, a bibliometric analysis on regenerative periodontics surgery identified a total of 422 authors [39]. Eighty-two percent of whom contributed to a single article, while 43 authors contributed to two articles each [39]. Another study showed that an average of 5.29 authors contributed to the most-cited articles on periodontics, but only five articles were found with a single author [44]. Asiri et al. demonstrated that among most-cited papers on dentistry, 20 articles were written by a single author, while 27 articles were written by two authors [45]. Nieri et al. [46] evaluated the citation of classic articles on periodontology and concluded that the most-cited articles had more authors in comparison with less-cited articles. It should be underscored that the same authors occupy the most impactful publication network, but the ranking may differ based on search strategy criteria.

The most cited authors in our study were: Cortellini, Zucchelli, Cairo, Nieri and De Sanctis, all of whom co-authored almost half of RCTs and accrued nearly 5000 citations (Table 2 and Table S1). All the above-mentioned authors are household names and come from Italy. These findings indicate that a small group of researchers provided the highest number of manuscripts, quite similarly to the other fields. Gutiérrez-Vela et al. [47] reported that 79.6% of the authors had contributed to just a single article. In our analysis Cortellini emerged as the most prolific author with 12 manuscripts, followed by Zucchelli with 10 articles. Cortellini was also the most productive author with 10 articles in the recent analysis on regenerative periodontics surgery [39]. In the bibliometric analysis of most-cited articles on bone grafting in dentistry, on the other hand, Buser had the highest number of citations (2648 citations, 12/100) [48]. It was the University of Bern that produced the largest number of articles (2565 citations, 13/100) [48].

These top one hundred most-cited RCTs encompass a wide range of topics with varying focus areas across different time periods. The most frequently referenced keywords were “root coverage” and “gingival recession”. By contrast, the most-researched themes in case of periodontics studies were “periodontology,” “implantology,” “oral biology” and “detection of bacteria” [47,49]. Our analysis revealed that the main domain of research was the type of graft, closely followed by GTR and EMD applications. The efficacy and cost-effectiveness of available therapeutic approaches have been a matter of extensive debate. Most studies were dedicated to assessing CTG/SCTG, whereas some others focused on ADMG and XCM. One article compared patient morbidity and root coverage outcomes with CTG or DGG and received the highest citation impact [14]. No differences were observed in the post-operative pain and clinical outcomes between groups. The publications on CTG were closely followed by an article comparing CTG to XCM [17], and another which focused on comparing CTG to ADM [18]. These two articles accumulated 167 and 160 citations, respectively. Both concluded that XCM and ADM may present a viable alternative to CTG, without the morbidity of soft tissue graft harvest. In our analysis 21 RCTs focused on GTR, among which 14 were dedicated to resorbable membranes, and 7 used non-resorbable membranes. It should be highlighted that this approach was popular in previous decades, and apart from one study, other RCTs were published from 1996 to 2005 [50]. It might be explained by the fact that GTR was associated with inherent technical difficulties and did not provide a significant advantage over CTG/SCTG [51]. GTR has undoubtedly lost its prominence in root coverage techniques over recent years. Furthermore, in many cases of GRs, a thin periodontal phenotype is often present, which constitutes a risk factor for recession progression. The legitimate goal in such cases is to modify thin phenotypes using CTG/SCTG, ADMG or XCM.

In our study, CAF, was the most frequently employed flap. This approach received decisively higher citation count when compared to TUN technique, which was the second popular surgical approach. The prevalent citation of CAF, especially in combination with CTG/SCTG, is due to the early popularity of this technique, which led to several modifications and further refinements [7]. Two studies received an impressive citation impact. Aichelmann-Reidy et al. [18] compared the recession reduction achieved between CAF sutured with tension and in CAF closed without tension. The authors concluded that the higher the flap tension, the lower the recession reduction 3 months after surgery. In the second study Zucchelli et al. [19] analyzed root coverage and esthetic outcomes of CAF with and without vertical releasing incisions in the treatment of multiple gingival recessions. Both mentioned articles might be considered hallmark papers in the field of periodontal plastic surgery. The high effectiveness of this treatment method compared to other techniques is also important [7,8]. Tunneling technique on the other hand is a relatively novel approach, which might have potentially impacted on the total citation score. It is reasonable to speculate that TUN technique will gain in citation in the future. Due to flap design lacking vertical incisions, TUN offers several benefits in terms of improved blood supply of the graft and reduced postoperative morbidity [52]. A high efficiency for TUN approach in terms of mean root coverage for multiple and single GRs was found in a systematic review, 87.87 ± 16.45% and 82.75 ± 19.7%, respectively [9]. Moreover, a recent network meta-analysis reported that TUN was associated with a significantly higher esthetic outcomes when compared to CAF [52]. However, among the one hundred most-cited trials in our analysis, less than 10 papers made use of TUN. For the same reason, areas of current trends (e.g., emerging regenerative therapies, recently introduced growth factors, minimally invasive techniques) may be underrepresented in the current analysis, and should be explored in the upcoming studies.

### Study Limitations

This study has some important limitations. Firstly, although a comprehensive search of the Web of Science database belonging to Clarivate was conducted, it was limited to English-language database. It should be underscored that the addition of other articles from different databases such as PubMed and Google Scholar might have given different datasets and findings. A lack of published studies in languages other than English might be due to systemic biases favoring English in scholarly communication. Secondly, there was a lack of gray literature database search. Moreover, due to time constraints on gathering citation data, some more recent studies might potentially have been excluded. However, it is highly unlikely for recently published RCTs to accumulate considerable number of citations. Thirdly, we did not distinguish the self-citations of authors that may inflate the ratio of citations. Although self-citations can be recognized as a limitation of this work, it should be noted that, the study search and selection was made by two researchers (B.G. and A.S.) not affiliated with Italian institutions. The analysis of self-citations with databases such as Scopus would further strengthen the methodological rigor. Quite interestingly, self-citation had a minor impact on common bibliometric measures in academic plastic surgery and did not change significantly between academic racks [53]. All of which, however, may cause potential biases to some extent (English-only, database choice, self-citations), but this study did not assess any biases or how they may affect reported observations. It is also worth emphasizing that highly cited studies may not necessarily represent the most rigorous or clinically relevant evidence. It should also be underscored that “most-cited” does not mean “most effective” and the sources of such information are other types of publications, i.e., systematic reviews and meta-analyses. It would be especially useful to include a critical appraisal (quality assessment) of the 100 RCTs, by using, for example, the Mixed Methods Appraisal Tool (MMAT) [54]. That said, assessing the quality of research falls more within the scope of statistical reviews and meta-analyses, which exceeds the scope of this work and bibliometric analysis. Other useful elements that could be included in the future studies are the impact factor (IF) of the journals, bibliometric indicators, and citation analysis (e.g., limitations of h-index, normalized citation metrics) and the number of references and the sample size. In our analysis, we applied parameters typical of bibliometric evaluation, which do not include aspects related to study design details such as sample size. Regarding IF, it is difficult to provide meaningful comparisons when the analysis covers studies published over a wide time span, during which journal IF values may have changed. It was also proposed to revise the formula of IF calculation and highlight the concept of the importance of individual articles which is dependent on factors such as ethical soundness of the journal guidelines, submission regulations, and editing processes [55]. Several factors that can affect IF rating [56]. Journals with open access may be more available for further citation. Conversely, highly specialized journals might gather less audience than general journals. The main problem with h-index is that it underestimates the ranking of scientists publishing papers receiving remarkably high citations and results in high values of A (the empirical proportionality constant A, called Hirsch constant) [57]. Since the h-index is not field-normalized, its value may be limited by the sheer size of the academic specialty [58]. The National Institutes of Health (NIH) developed a new metric called the relative citation ratio (RCR), which improved upon the weakness of the h-index and more accurately reflected research impact [59].

All things considered, conducting bibliometric analysis that includes a diverse dataset would be of certain benefit. It might also be remarkably interesting to evaluate the quality of 100-most cited RCTs and the citation impact of different study designs. Nonetheless, within its limitations, our study provides valuable insights into the trends and influential research in the field of gingival recession treatment.

## 5. Conclusions

This bibliometric analysis identified the one hundred most-cited clinical trials on gingival recession treatment. It showed dominance of Italian research groups and focused on CAF and CTG/SCTG procedures. While these findings illustrate research influence and collaboration networks, citation frequency should not be equated with clinical superiority. Future work should combine bibliometric mapping with critical quality appraisal and explore whether citation trends align with best available evidence.

## 6. Practical Implications

As RTCs have the highest scientific input, our analysis may represent a fundamental to summarizing relevant data regarding evidence based gingival recessions coverage procedures and demonstrate the future map road for research. Moreover, nowadays, living in the dogma of time constrains, our analysis enables practitioners to become acquainted with the most cited high-quality articles in the field.

## Figures and Tables

**Table 1:** Top 25 most-cited publications.

Authors	Country	Article Title	Journal Title	Year	Volume, Issue	Citation Number
Zucchelli, G.; Mele, M.; Stefanini, M.; Mazzotti, C.; Marzadori, M.; Montebugnoli, L.; de Sanctis, M. [14]	Italy	Patient morbidity and root coverage outcome after subepithelial connective tissue and de-epithelialized grafts: a comparative randomized-controlled clinical trial	*Journal of Clinical Periodontology*	2010	37, 8	284
Aroca, S.; Keglevich, T.; Barbieri, B.; Gera, I.; Etienne, D. [15]	France	Clinical evaluation of a modified coronally advanced flap alone or in combination with a platelet-rich fibrin membrane for the treatment of adjacent multiple gingival recessions: a 6-month study	*Journal of Periodontology*	2009	80, 2	184
Prato, G.P.; Pagliaro, U.; Baldi, C.; Nieri, M.; Saletta, D.; Cairo, F.; Cortellini, P. [16]	Italy	Coronally advanced flap procedure for root coverage. Flap with tension versus flap without tension: A randomized controlled clinical study	*Journal of Periodontology*	2000	71, 2	172
McGuire, M.K.; Scheyer, E.T. [17]	the USA	Xenogeneic collagen matrix with coronally advanced flap compared to connective tissue with coronally advanced flap for the treatment of dehiscence-type recession defects	*Journal of Periodontology*	2010	81, 8	167
Aichelmann-Reidy, M.E.; Yukna, R.A.; Evans, G.H.; Nasr, H.F.; Mayer, E.T. [18]	the USA	Clinical evaluation of acellular allograft dermis for the treatment of human gingival recession	*Journal of Periodontology*	2001	72, 8	160
Zucchelli, G.; Mele, M.; Mazzotti, C.; Marzadori, M.; Montebugnoli, L.; De Sanctis, M. [19]	Italy	Coronally advanced flap with and without vertical releasing incisions for the treatment of multiple gingival recessions: a comparative controlled randomized clinical trial	*Journal of Periodontology*	2009	80, 7	157
Cortellini, P.; Tonetti, M.; Baldi, C.; Francetti, L.; Rasperini, G.; Rotundo, R.; Nieri, M.; Franceschi, D.; Labriola, A.; Prato, G.P. [20]	Italy	Does placement of a connective tissue graft improve the outcomes of coronally advanced flap for coverage of single gingival recessions in upper anterior teeth? A multi-center, randomized, double-blind, clinical trial	*Journal of Clinical Periodontology*	2009	36, 1	155
Aroca, S.; Keglevich, T.; Nikolidakis, D.; Gera, I.; Nagy, K.; Azzi, R.; Etienne, D. [21]	France	Treatment of class III multiple gingival recessions: a randomized-clinical trial	*Journal of Clinical Periodontology*	2010	37, 1	152
Aroca, S.; Molnár, B.; Windisch, P.; Gera, I.; Salvi, G.E.; Nikolidakis, D.; Sculean, A. [22]	France	Treatment of multiple adjacent Miller class I and II gingival recessions with a Modified Coronally Advanced Tunnel (MCAT) technique and a collagen matrix or palatal connective tissue graft: a randomized, controlled clinical trial	*Journal of Clinical Periodontology*	2013	40, 1	151
Zucchelli, G.; Amore, C.; Sforza, N.M.; Montebugnoli, L.; De Sanctis, M. [23]	Italy	Bilaminar techniques for the treatment of recession-type defects. A comparative clinical study	*Journal of Clinical Periodontology*	2003	30, 10	151
Pini-Prato, G.P.; Cairo, F.; Nieri, M.; Franceschi, D.; Rotundo, R.; Cortellini, P. [24]	Italy	Coronally advanced flap versus connective tissue graft in the treatment of multiple gingival recessions: a split-mouth study with a 5-year follow-up	*Journal of Clinical Periodontology*	2010	37, 7	138
Burkhardt, R.; Lang, N.P. [25]	Switzerland	Coverage of localized gingival recessions: comparison of micro- and macrosurgical techniques	*Journal of Clinical Periodontology*	2005	32, 3	138
Bouchard, P.; Etienne, D.; Ouhayoun, J.P.; Nilveus, R. [26]	France	Subepithelial connective-tissue grafts in the treatment of gingival recessions-a comparative-study of 2 procedures	*Journal of Periodontology*	1994	65, 10	137
Jepsen, K.; Jepsen, S.; Zucchelli, G.; Stefanini, M.; de Sanctis, M.; Baldini, N.; Greven, B.; Heinz, B.; Wennström, J.; Cassel, B.; Vignoletti, F.; Sanz, M. [27]	Germany	Treatment of gingival recession defects with a coronally advanced flap and a xenogeneic collagen matrix: a multicenter randomized clinical trial	*Journal of Clinical Periodontology*	2013	40, 1	131
da Silva, R.C.; Joly, J.C.; de Lima, A.F.M.; Tatakis, D.N. [28]	Brazil	Root coverage using the coronally positioned flap with or without a subepithelial connective tissue graft	*Journal of Periodontology*	2004	75, 3	125
Cummings, L.C.; Kaldahl, W.B.; Allen, E.P. [29]	the USA	Histologic evaluation of autogenous connective tissue and acellular dermal matrix grafts in humans	*Journal of Periodontology*	2005	76, 2	123
McGuire, M.K.; Nunn, M. [30]	the USA	Evaluation of human recession defects treated with coronally advanced flaps and either enamel matrix derivative or connective tissue. Part 1: Comparison of clinical parameters	*Journal of Periodontology*	2003	74, 8	118
Jahnke, P.Y.; Sandifer, J.B.; Gher, M.E.; Gray, J.L.; Richardson, A.C. [31]	the USA	Thick free gingival and connective-tissue autografts for root coverage	*Journal of Periodontology*	1993	64, 4	117
Paolantonio, M.; Dolci, M.; Esposito, P.; D’Archivio, D.; Lisanti, L.; Di Luccio, A.; Perinetti, G. [32]	Italy	Subpedicle acellular dermal matrix graft and autogenous connective tissue graft in the treatment of gingival recessions: A comparative 1-year clinical study	*Journal of Periodontology*	2002	73, 11	116
Roccuzzo, M.; Lungo, M.; Corrente, G.; Gandolfo, S. [33]	Italy	Comparative study of a bioresorbable and a non-resorbable membrane in the treatment of human buccal gingival recessions	*Journal of Periodontology*	1996	67, 1	112
Harris, R.J. [34]	the USA	A comparative study of root coverage obtained with guided tissue regeneration utilizing a bioabsorbable membrane versus the connective tissue with partial-thickness double pedicle graft	*Journal of Periodontology*	1997	68, 8	108
Zucchelli, G.; Clauser, C.; De Sanctis, M.; Calandriello, M. [35]	Italy	Mucogingival versus guided tissue regeneration procedures in the treatment of deep recession type defects	*Journal of Periodontology*	1998	69, 2	107
Tal, H.; Moses, O.; Zohar, R.; Meir, H.; Nemcovsky, C. [36]	Israel	Root coverage of advanced gingival recession: A comparative study between acellular dermal matrix allograft and subepithelial connective tissue grafts	*Journal of Periodontology*	2002	73, 12	103
Cardaropoli, D.; Tamagnone, L.; Roffredo, A.; Gaveglio, L. [37]	Italy	Treatment of gingival recession defects using coronally advanced flap with a porcine collagen matrix compared to coronally advanced flap with connective tissue graft: a randomized controlled clinical trial	*Journal of Periodontology*	2012	83, 3	101
Tonetti, M.S.; Cortellini, P.; Pellegrini, G.; Nieri, M.; Bonaccini, D.; Allegri, M.; Bouchard, P.; Cairo, F.; Conforti, G.; Fourmousis, I.; Graziani, F.; Guerrero, A.; Halben, J.; Malet, J.; Rasperini, G.; Topoll, H.; Wachtel, H.; Wallkamm, B.; Zabalegui, I.; Zuhr, O. [38]	Italy	Xenogenic collagen matrix or autologous connective tissue graft as adjunct to coronally advanced flaps for coverage of multiple adjacent gingival recession: Randomized trial assessing non-inferiority in root coverage and superiority in oral health-related quality of life	*Journal of Clinical Periodontology*	2018	45, 1	93

**Table 2:** Top five productive and cited authors.

Author	Papers		Citations		Citations/Paper	
	*n*	Rank	*n*	Rank	Rank	
Cortellini, Pierpaolo	12	1	1112	2	92.7	4
Zucchelli, Giovanni	10	2	1174	1	117.4	2
Cairo, Francesco	9	3	830	5	92.2	5
Nieri, Michele	9	3	952	3	105.8	3
De Sanctis, Massimo	6	5	881	4	146.8	1

**Table 3:** Top 10 universities with the highest number of papers and citations.

University	Papers	Citations	Citations/Paper
Univ Bologna	10	1174	117.4
Univ Florence	10	996	99.6
Univ Estadual Campinas	9	610	67.8
Univ Siena	8	1021	127.6
Univ Milan	6	473	78.8
Univ Michigan	6	400	66.7
Univ Bern	5	561	112.2
Univ G Dannunzio	4	342	85.5
Univ Turin	4	315	78.8
Univ Sao Paulo	4	258	64.5

**Table 4:** Top 10 countries with the highest number of papers and citations.

Country	Papers	Citations	Citations/Papers
Italy	36	3386	94.1
USA	28	2165	77.3
Brazil	17	1087	63.9
Switzerland	13	1282	98.6
Germany	11	796	72.4
France	7	759	108.4
Sweden	6	529	88.2
Türkiye	5	275	55.0
Hungary	3	487	162.3
Spain	3	299	99.7

**Table 5:** Journals with the highest number of papers and citations.

Journal	Papers	Citations	Citations/Paper
*Journal of Periodontology*	59	4939	83.7
*Journal of Clinical Periodontology*	37	3224	87.1
*International Journal of Periodontics & Restorative Dentistry*	2	94	47.0
*Clinical Oral Investigations*	1	64	64.0
*Implant Dentistry*	1	47	47.0

**Table 6:** Top 20 keywords.

Keyword	Occurrences	Total Link Strength (VOSviewer)
Root coverage	60	233
Gingival recession	42	147
Defects	32	137
Gingival recession/surgery	28	153
Guided tissue regeneration	26	117
Surgical flaps	25	129
Mucogingival surgery	21	106
Coronally advanced flap	20	57
Connective-tissue graft	18	73
Gingival recession/therapy	18	117
Regeneration	18	89
Double pedicle graft	17	105
Bioabsorbable membrane	16	103
Comparison studies	16	100
Connective-tissue	16	85
Thickness	16	73
Grafts, connective tissue	15	85
Positioned flap	15	58
Tooth root	12	68
Periodontal regeneration	11	57

**Table 7:** Main research topics associated with flap design and biomaterials (if used).

Main Research Domain	Comparison	Flap Design	Biomaterials
**Type of graft (*n* = 34)**	CTG vs. ADMG (*n* = 10) CTG vs. flap alone (*n* = 7) CTG vs. XCM (*n* = 7) ADMG vs. flap alone (*n* = 4) XCM vs. flap alone (*n* = 2) CTG vs. FGG (*n* = 2) ADMG with basement side vs. ADMG with connective tissue side against the root (*n* = 1) CTG vs. HF-DDS (*n* = 1)	CAF (*n* = 10) CAF (*n* = 7) CAF (*n* = 4) TUN (*n* = 2) repositioned flap (*n* = 1) CAF (*n* = 4) CAF (*n* = 2) CAF (*n* = 1) repositioned flap (*n* = 1) CAF (*n* = 1) CAF (*n* = 1)	CTG vs. ADMG (*n* = 10) CTG (*n* = 7) CTG vs. XCM (*n* = 7) ADMG (*n* = 4) XCM (*n* = 2) CTG vs. FGG (*n* = 2) ADMG (*n* = 1) CTG vs. HF-DDS (*n* = 1)
**GTR (*n* = 17)**	RM vs. CTG (*n* = 5) RM vs. NRM (*n* = 3) RM vs. flap alone (*n* = 2) NRM vs. CTG (*n* = 2) NRM vs. FGG (*n* = 1) RM + DFDBA vs. CTG (*n* = 1) RM + DFDBA vs. RM (*n* = 1) RM vs. CPRT vs. CTG (*n* = 1) RM vs. modified envelope technique (*n* = 1)	CAF (*n* = 5) CAF (*n* = 2) Double pedicle flap (*n* = 1) CAF (*n* = 2) CAF (*n* = 2) CAF (*n* = 1) CAF (*n* = 1) CAF (*n* = 1) CAF (*n* = 1) CAF vs. modified envelope technique (*n* = 1)	RM vs. CTG (*n* = 5) RM vs. NRM (*n* = 3) RM (*n* = 2) NRM vs. CTG (*n* = 2) NRM vs. FGG (*n* = 1) RM + DFDBA vs. CTG. (*n* = 1) RM + DFDBA vs. RM (*n* = 1) RM vs. CPRT vs. CTG (*n* = 1) RM vs. CTG (*n* = 1)
**EMD (*n* = 13)**	EMD vs. flap alone (*n* = 7) EMD vs. CTG (*n* = 3) EMD + CTG vs. CTG (*n* = 2) EMD + CTG vs. EMD (*n* = 1)	CAF (*n* = 7) CAF (*n* = 3) CAF (*n* = 1) TUN (*n* = 1) CAF (*n* = 1)	EMD (*n* = 7) EMD vs. CTG (*n* = 3) EMD + CTG vs. CTG (*n* = 2) EMD + CTG vs. EMD (*n* = 1)
**Flap design (*n* = 9)**	TUN + CTG vs. CAF + CTG (*n* = 3) TUN + ADMG vs. CAF + ADMG (*n* = 2) CAF with vertical incisions vs. CAF without vertical incisions (*n* = 1) ADMG + CAF with vertical incisions vs. ADMG + CAF without vertical incisions (*n* = 1) TUN + CTG vs. CAF + EMD (*n* = 1) SCPF vs. CAF + CTG (*n* = 1)	TUN vs. CAF (*n* = 3) TUN vs. CAF (*n* = 2) CAF (*n* = 1) CAF (*n* = 1) TUN vs. CAF (*n* = 1) SCPF vs. CAF (*n* = 1)	CTG (*n* = 3) ADMG (*n* = 2) ADMG (*n* = 1) CTG vs. EMD (*n* = 1) SCPF vs. CAF + CTG (*n* = 1)
**Growth factors (*n* = 6)**	PRP vs. flap alone (*n* = 1) PRP + CTG vs. CTG (*n* = 1) PRF vs. flap alone (*n* = 1) PRF vs. CTG (*n* = 1) PCG vs. CTG (*n* = 1) CGF vs. flap alone (*n* = 1)	CAF (*n* = 1) CAF (*n* = 1) CAF (*n* = 1) CAF (*n* = 1) CAF (*n* = 1) CAF (*n* = 1)	PRP (*n* = 1) PRP + CTG. vs. CTG (*n* = 1 PRF (*n* = 1) PRF (*n* = 1) PCG vs. CTG (*n* = 1) CGF (*n* = 1)
**Treatment of root surface (*n* = 6)**	Root planning vs. root polishing (*n* = 2) Tetracycline + CTG vs. citric acid +CTG (*n* = 1) Tetracycline + fibrin glue vs. tetracycline (*n* = 1) TTC + FFFS + NRM vs. NRM (*n* = 1) Citric acid + CTG vs. CTG (*n* = 1)	CAF (*n* = 2) CAF (*n* = 1) CAF (*n* = 1) CAF (*n* = 1) CAF (*n* = 1)	CTG (*n* = 1) NRM (*n* = 1) CTG (*n* = 1)
**Type of CTG (*n* = 5)**	CTG vs. ECTG (*n* = 2) CTG vs. DGG (*n* = 1) CTG big, thick, positioned at CEJ vs. CTG small, thin, positioned apically to CEJ (*n* = 1) CTG of thickness ≥ 2 and height >4 mm vs. CTG of thickness <2 mm and height =4 mm (*n* = 1)	CAF (*n* = 2) CAF (*n* = 1) CAF (*n* = 1) CAF (*n* = 1)	CTG (*n* = 2) CTG (*n* = 1) CTG (*n* = 1) CTG (*n* = 1)
**NCCL restoration (*n* = 4)**	NCCL restoration before CAF vs. no NCCL restoration before CAF (*n* = 3) NCCL restoration with RMGI vs. NCCL restoration with MRC (*n* = 1)	CAF (*n* = 3) CAF (*n* = 1)	
**Others (*n* = 6)**	Surgical treatment vs. observation (*n* = 1) LST removal vs. flap alone (*n* = 1) CAF in smokers vs. non-smokers (*n* = 1) CAF + CTG in smokers. vs. non-smokers (*n* = 1) Microsurgical vs. non-microsurgical approach Flap suturing with tension vs. passive flap suturing (*n* = 1)	Double pedicle flap (*n* = 1) CAF (*n* = 1) CAF (*n* = 1) CAF (*n* = 1) Double pedicle flap (*n* = 1) CAF (*n* = 1)	CTG (*n* = 1) CTG (*n* = 1)

ADMG—acellular dermal matrix graft; CAF—coronally advanced flap; CEJ—cemento-enamel junction; CGF—concentrated growth factor; CPRT—combined periodontal regenerative technique (collagen membrane and collagen-incorporated hydroxyapatite); CTG—connective tissue graft; DFDBA—demineralized freeze-dried bone allograft LST—labial submucosal tissue; DGG—de-epithelialized gingival graft; ECTG—connective tissue graft with epithelial collar; EMD—enamel matrix derivative; FFSS—fibrin-fibronectin sealing system; FGG—free gingival graft; HF-DDS—a living human fibroblast-derived substitute; LST—labial submucosal tissue; PCG—plate-let concentrate graft; PRF—platelet-rich plasma; PRP—platelet-rich fibrin; MCAT—modified coronally advanced tunnel; MRC—microfilled resin composite; NCCL—non-carious cervical lesion; NRM—non-resorbable membrane; RM—resorbable membrane; RMGI—resin modified glass ionomer cement; SCPF—semilunar coronally positioned flap; TTC—tetracycline HCL; TUN—tunnel technique; XCM—xenogeneic collagen matrix.

## Data Availability

No new data were created or analyzed in this study. Data sharing is not applicable to this article.

## Supplementary Materials

The following supporting information can be downloaded at: https://www.mdpi.com/article/10.3390/jfb16100364/s1, Table S1: The 100 most-cited clinical trials on gingival recession treatment.
