# Supplementary material for: Bibliometric Analysis of the 100 Most-Cited Clinical Trials on Gingival Recession Treatment: Trends in Flap Design, Biomaterials, and Global Contributions
Source: J Funct Biomater. 2025 Oct 1;16(10):364. doi: 10.3390/jfb16100364 (PMC12565512; doi:10.3390/jfb16100364)
Supplement: Supplementary file 1 [file jfb-16-00364-s001.zip › jfb-3826589-supplementary.pdf]

Table S1 The 100 most-cited clinical trials on gingival recession treatment.

	A	B	C	D	E	F	G	H	I	J	K
1	Title	Authors	Source Title	Publicatio	Volume	Issue	Beginning	Ending Pa	DOI	Total Citations	Average per Year
2	Patient morbidity and root coverage outcome after subepithelial connective tissue and de	Zucchelli, Giovanni; Mele, Monica; Stefanini, M	JOURNAL OF CLINICAL PERIODONTOLOGY	2010	37	8	728	738	10.1111/j.1600-051X.2010.01550.x	284	18,93
3	Clinical Evaluation of a Modified Coronally Advanced Flap Alone or in Combination With a	Aroca, Sofia; Keglevich, Tibor; Barbieri, Bruno; (	JOURNAL OF PERIODONTOLOGY	2009	80	2	244	252	10.1902/jop.2009.080253	184	11,5
4	Coronally advanced flap procedure for root coverage. Flap with tension versus flap withou	Prato, GP; Pagliaro, U; Baldi, C; Nieri, M; Saletta	JOURNAL OF PERIODONTOLOGY	2000	71	2	188	201	10.1902/jop.2000.71.2.188	172	6,88
5	Xenogeneic Collagen Matrix With Coronally Advanced Flap Compared to Connective Tissu	McGuire, Michael K.; Scheyer, E. Todd	JOURNAL OF PERIODONTOLOGY	2010	81	8	1108	1117	10.1902/jop.2010.090698	167	11,13
6	Clinical evaluation of acellular allograft dermis for the treatment of human gingival recessi	Aichelmann-Reidy, ME; Yukna, RA; Evans, GH; N	JOURNAL OF PERIODONTOLOGY	2001	72	8	998	1005	10.1902/jop.2001.72.8.998	160	6,67
7	Coronally Advanced Flap With and Without Vertical Releasing Incisions for the Treatment	Zucchelli, G.; Mele, M.; Mazzotti, C.; Marzadori, M	JOURNAL OF PERIODONTOLOGY	2009	80	7	1083	1094	10.1902/jop.2009.090041	157	9,81
8	Does placement of a connective tissue graft improve the outcomes of coronally advanced	Cortellini, Pierpaolo; Tonetti, Maurizio; Baldi, C	JOURNAL OF CLINICAL PERIODONTOLOGY	2009	36	1	68	79	10.1111/j.1600-051X.2008.01346.x	155	9,69
9	Treatment of class III multiple gingival recessions: a randomized-clinical trial	Aroca, Sofia; Keglevich, Tibor; Nikolidakis, Dim	JOURNAL OF CLINICAL PERIODONTOLOGY	2010	37	1	88	97	10.1111/j.1600-051X.2009.01492.x	152	10,13
10	Treatment of multiple adjacent Miller class I and II gingival recessions with a Modified Cor	Aroca, Sofia; Molnar, Balint; Windisch, Peter; G	JOURNAL OF CLINICAL PERIODONTOLOGY	2013	40	7	713	720	10.1111/jcpe.12112	151	12,58
11	Bilaminar techniques for the treatment of recession-type defects. A comparative clinical s	Zucchelli, G; Amore, C; Sforza, NM; Montebugn	JOURNAL OF CLINICAL PERIODONTOLOGY	2003	30	10	862	870	10.1034/j.1600-051X.2003.00397.x	151	6,86
12	Coronally advanced flap <i>versus</i> connective tissue graft in the treatment of multiple	Pini-Prato, Giovan Paolo; Cairo, Francesco; Nie	JOURNAL OF CLINICAL PERIODONTOLOGY	2010	37	7	644	650	10.1111/j.1600-051X.2010.01559.x	138	9,2
13	Coverage of localized gingival recessions: comparison of micro- and macrosurgical techni	Burkhardt, R; Lang, NP	JOURNAL OF CLINICAL PERIODONTOLOGY	2005	32	3	287	293	10.1111/j.1600-051X.2005.00660.x	138	6,9
14	SUBEPITHELIAL CONNECTIVE-TISSUE GRAFTS IN THE TREATMENT OF GINGIVAL RECESSI	BOUCHARD, P; ETIENNE, D; OUHAYOUN, JP; NI	JOURNAL OF PERIODONTOLOGY	1994	65	10	929	936	10.1902/jop.1994.65.10.929	137	4,42
15	Treatment of gingival recession defects with a coronally advanced flap and a xenogeneic c	Jepsen, Karin; Jepsen, Soren; Zucchelli, Giovan	JOURNAL OF CLINICAL PERIODONTOLOGY	2013	40	1	82	89	10.1111/jcpe.12019	131	10,92
16	Root coverage using the coronally positioned flap with or without a subepithelial connect	da Silva, RC; Joly, JC; de Lima, AFM; Tatakis, DN	JOURNAL OF PERIODONTOLOGY	2004	75	3	413	419	10.1902/jop.2004.75.3.413	125	5,95
17	Histologic evaluation of autogenous connective tissue and acellular dermal matrix grafts i	Cummings, LC; Kaldahl, WB; Allen, EP	JOURNAL OF PERIODONTOLOGY	2005	76	2	178	186	10.1902/jop.2005.76.2.178	123	6,15
18	Evaluation of human recession defects treated with coronally advanced flaps and either e	McGuire, MK; Nunn, M	JOURNAL OF PERIODONTOLOGY	2003	74	8	1110	1125	10.1902/jop.2003.74.8.1110	118	5,36
19	THICK FREE GINGIVAL AND CONNECTIVE-TISSUE AUTOGRAFTS FOR ROOT COVERAGE	JAHNKE, PV; SANDIFER, JB; GHER, ME; GRAY, JL	JOURNAL OF PERIODONTOLOGY	1993	64	4	315	322	10.1902/jop.1993.64.4.315	117	3,66
20	Subpedicle acellular dermal matrix graft and autogenous connective tissue graft in the tre	Paolantonio, M; Dolci, M; Esposito, P; D'Archiv	JOURNAL OF PERIODONTOLOGY	2002	73	11	1299	1307	10.1902/jop.2002.73.11.1299	116	5,04
21	Comparative study of a bioresorbable and a non-resorbable membrane in the treatment o	Roccuzzo, M; Lungo, M; Corrente, G; Gandolfo,	JOURNAL OF PERIODONTOLOGY	1996	67	1	7	14	10.1902/jop.1996.67.1.7	112	3,86
22	A comparative study of root coverage obtained with guided tissue regeneration utilizing a	Harris, RJ	JOURNAL OF PERIODONTOLOGY	1997	68	8	779	790	10.1902/jop.1997.68.8.779	108	3,86
23	Mucogingival versus guided tissue regeneration procedures in the treatment of deep reces	Zucchelli, G; Clauser, C; De Sanctis, M; Calandr	JOURNAL OF PERIODONTOLOGY	1998	69	2	138	145	10.1902/jop.1998.69.2.138	107	3,96
24	Root coverage of advanced gingival recession: A comparative study between acellular derr	Tal, H; Moses, O; Zohar, R; Meir, H; Nemcovsky,	JOURNAL OF PERIODONTOLOGY	2002	73	12	1405	1411	10.1902/jop.2002.73.12.1405	103	4,48
25	Treatment of Gingival Recession Defects Using Coronally Advanced Flap With a Porcine C	Cardaropoli, Daniele; Tamagnone, Lorenzo; Ro	JOURNAL OF PERIODONTOLOGY	2012	83	3	321	328	10.1902/jop.2011.110215	101	7,77
26	Xenogenic collagen matrix or autologous connective tissue graft as adjunct to coronally a	Tonetti, Maurizio S.; Cortellini, Pierpaolo; Pelle	JOURNAL OF CLINICAL PERIODONTOLOGY	2018	45	1	78	88	10.1111/jcpe.12834	93	13,29
27	The effect of platelet-rich plasma on the coronally advanced flap root coverage procedure:	Huang, LH; Neiva, REF; Soehren, SE; Giannobil	JOURNAL OF PERIODONTOLOGY	2005	76	10	1768	1777	10.1902/jop.2005.76.10.1768	93	4,65
28	The clinical effect of acellular dermal matrix on gingival thickness and root coverage comp	Woodyard, JG; Greenwell, H; Hill, M; Drisko, C; I	JOURNAL OF PERIODONTOLOGY	2004	75	1	44	56	10.1902/jop.2004.75.1.44	93	4,43
29	Comparison of 2 clinical techniques for treatment of gingival recession	Wang, HL; Bunyaratavej, P; Labadie, M; Shyr, Y;	JOURNAL OF PERIODONTOLOGY	2001	72	10	1301	1311	10.1902/jop.2001.72.10.1301	93	3,88
30	Predictable multiple site root coverage using an acellular dermal matrix allograft	Henderson, RD; Greenwell, H; Drisko, C; Regen	JOURNAL OF PERIODONTOLOGY	2001	72	5	571	582	10.1902/jop.2001.72.5.571	93	3,88
31	Subpedicle connective tissue graft versus free gingival graft in the coverage of exposed roo	Paolantonio, M; diMurro, C; Cattabriga, A; Catt	JOURNAL OF CLINICAL PERIODONTOLOGY	1997	24	1	51	56	10.1111/j.1600-051X.1997.tb01184	92	3,29
32	Guided tissue regeneration versus mucogingival surgery in the treatment of human bucca	Prato, GP; Clauser, C; Cortellini, P; Tinti, C; Vin	JOURNAL OF PERIODONTOLOGY	1996	67	11	1216	1223	10.1902/jop.1996.67.11.1216	92	3,17
33	Xenogeneic collagen matrix versus connective tissue graft for buccal soft tissue augment	Cairo, Francesco; Barbato, Luigi; Tonelli, Paolo	JOURNAL OF CLINICAL PERIODONTOLOGY	2017	44	7	769	776	10.1111/jcpe.12750	90	11,25
34	Coronally advanced flap procedure for root coverage. Treatment of root surface: Root plar	Pini-Prato, G; Baldi, C; Pagliaro, U; Nieri, M; Sale	JOURNAL OF PERIODONTOLOGY	1999	70	9	1064	1076	10.1902/jop.1999.70.9.1064	87	3,35
35	Clinical efficacy of coronally advanced flap with or without connective tissue graft for the	Cairo, Francesco; Cortellini, Pierpaolo; Pilloni,	JOURNAL OF CLINICAL PERIODONTOLOGY	2016	43	10	849	856	10.1111/jcpe.12590	85	9,44
36	Comparative study of Emdogain® and coronally advanced flap technique in the treatment	Hägewald, S; Spahr, A; Rompola, E; Haller, B; H	JOURNAL OF CLINICAL PERIODONTOLOGY	2002	29	1	35	41	10.1034/j.1600-051x.2002.290106.x	85	3,7
37	Coronally advanced flap for the treatment of buccal gingival recessions with and without	Modica, F; Del Pizzo, M; Roccuzzo, M; Romagno	JOURNAL OF PERIODONTOLOGY	2000	71	11	1693	1698	10.1902/jop.2000.71.11.1693	84	3,36
38	Tunnel technique with connective tissue graft versus coronally advanced flap with ename	Zuhr, Otto; Rebele, Stephan F.; Schneider, Davi	JOURNAL OF CLINICAL PERIODONTOLOGY	2014	41	6	582	592	10.1111/jcpe.12178	82	7,45
39	Gingival recession treatment: Guided tissue regeneration with bioabsorbable membrane	Tatakis, DN; Trombelli, L	JOURNAL OF PERIODONTOLOGY	2000	71	2	299	307	10.1902/jop.2000.71.2.299	81	3,24
40	Does the dimension of the graft influence patient morbidity and root coverage outcomes?	Zucchelli, Giovanni; Mounssif, Ilham; Mazzotti,	JOURNAL OF CLINICAL PERIODONTOLOGY	2014	41	7	708	716	10.1111/jcpe.12256	80	7,27
41	Coronally advanced flap with and without connective tissue graft for the treatment of mul	Zucchelli, Giovanni; Mounssif, Ilham; Mazzotti,	JOURNAL OF CLINICAL PERIODONTOLOGY	2014	41	4	396	403	10.1111/jcpe.12224	80	7,27
42	A comparative study of root coverage with connective tissue and platelet concentrate graf	Cheung, WS; Griffin, TJ	JOURNAL OF PERIODONTOLOGY	2004	75	12	1678	1687	10.1902/jop.2004.75.12.1678	79	3,76
43	Comparative 6-month clinical study of a subepithelial connective tissue graft and acellula	Novaes, AB; Grisi, DC; Molina, GO; Souza, SLS;	JOURNAL OF PERIODONTOLOGY	2001	72	11	1477	1484	10.1902/jop.2001.72.11.1477	79	3,29
44	Treatment of gingival recession: Comparative study between subepithelial connective tiss	Rosetti, EP; Marcantonio, RAC; Rossa, C; Chav	JOURNAL OF PERIODONTOLOGY	2000	71	9	1441	1447	10.1902/jop.2000.71.9.1441	78	3,12
45	Coronally positioned flap plus resin-modified glass ionomer restoration for the treatment	Santamaria, Mauro Pedrine; Suaid, Fabricia Fe	JOURNAL OF PERIODONTOLOGY	2008	79	4	621	628	10.1902/jop.2008.070285	77	4,53
46	Coronally advanced flap with and without connective tissue graft for the treatment of sing	Cairo, Francesco; Cortellini, Pierpaolo; Tonetti	JOURNAL OF CLINICAL PERIODONTOLOGY	2012	39	8	760	768	10.1111/j.1600-051X.2012.01903.x	76	5,85
47	Subpedicle connective tissue graft versus guided tissue regeneration with bioabsorbable	Trombelli, L; Scabbia, A; Tatakis, DN; Calura, G	JOURNAL OF PERIODONTOLOGY	1998	69	11	1271	1277	10.1902/jop.1998.69.11.1271	76	2,81
48	Acellular dermal matrix and coronally advanced flap or tunnel technique in the treatment c	Tavelli, Lorenzo; Barootchi, Shayan; Di Gianfilip	JOURNAL OF CLINICAL PERIODONTOLOGY	2019	46	9	937	948	10.1111/jcpe.13163	74	12,33
49	Enamel matrix derivative and coronal flaps to cover marginal tissue recessions	Castellanos, Adriana; de la Rosa, Manuel; de la	JOURNAL OF PERIODONTOLOGY	2006	77	1	7	14	10.1902/jop.2006.77.1.7	74	3,89
50	Root coverage with a coronally positioned flap used in combination with enamel matrix de	Pilloni, Andrea; Paolantonio, Michele; Camarg	JOURNAL OF PERIODONTOLOGY	2006	77	12	2031	2039	10.1902/jop.2006.050390	73	3,84
51	Tunnel technique with connective tissue graft versus coronally advanced flap with ename	Rebele, Stephan F.; Zuhr, Otto; Schneider, Davi	JOURNAL OF CLINICAL PERIODONTOLOGY	2014	41	6	593	603	10.1111/jcpe.12254	72	6,55
52	Coronally advanced flap with or without enamel matrix derivative for root coverage: a 2-yea	Del Pizzo, M; Zucchelli, G; Modica, F; Villa, R; De	JOURNAL OF CLINICAL PERIODONTOLOGY	2005	32	11	1181	1187	10.1111/j.1600-051X.2005.00831.x	72	3,6
53	Comparative clinical study of a bioabsorbable membrane and subepithelial connective tis	Borghetti, A; Glise, JM; Monnet-Corti, V; Dejou,	JOURNAL OF PERIODONTOLOGY	1999	70	2	123	130	10.1902/jop.1999.70.2.123	68	2,62
54	Connective tissue graft plus resin-modified glass ionomer restoration for the treatment of	Santamaria, Mauro Pedrine; Bovi Ambrosano, (	JOURNAL OF CLINICAL PERIODONTOLOGY	2009	36	9	791	798	10.1111/j.1600-051X.2009.01441.x	66	4,13
55	Treatment of gingival recession with titanium reinforced barrier membranes versus conne	Jepsen, K; Heinz, B; Halben, JH; Jepsen, S	JOURNAL OF PERIODONTOLOGY	1998	69	3	383	391	10.1902/jop.1998.69.3.383	66	2,44
56	Platelet-rich fibrin in the treatment of localized gingival recessions: a split-mouth randomi	Eren, Gulnihal; Atilla, Gul	CLINICAL ORAL INVESTIGATIONS	2014	18	8	1941	1948	10.1007/s00784-013-1170-5	65	5,91
57	Fibrin glue application in conjunction with tetracycline root conditioning and coronally pc	Trombelli, L; Scabbia, A; Wikesjo, UME; Calura,	JOURNAL OF CLINICAL PERIODONTOLOGY	1996	23	9	861	867	10.1111/j.1600-051X.1996.tb00624	65	2,24
58	Growth Factor-Mediated Treatment of Recession Defects: A Randomized Controlled Trial	McGuire, Michael K.; Scheyer, E. Todd; Schupb	JOURNAL OF PERIODONTOLOGY	2009	80	4	550	564	10.1902/jop.2009.080502	64	4
59	Use of platelet gel with connective tissue grafts for root coverage: a randomized-controlle	Keceli, Huseyin Gencay; Sengun, Dilek; Berberc	JOURNAL OF CLINICAL PERIODONTOLOGY	2008	35	3	255	262	10.1111/j.1600-051X.2007.01181.x	64	3,76
60	A two-year prospective study of coronally positioned flap with or without acellular dermal	de Queiroz Cortes, Antonieta; Sallum, Antonio	JOURNAL OF CLINICAL PERIODONTOLOGY	2006	33	9	683	689	10.1111/j.1600-051X.2006.00969.x	63	3,32
61	Coverage of Miller class I and II recession defects using enamel matrix proteins versus cor	Spahr, A; Haegewald, S; Tsoulfidou, F; Rompol	JOURNAL OF PERIODONTOLOGY	2005	76	11	1871	1880	10.1902/jop.2005.76.11.1871	63	3,15
62	Coronally positioned flap procedures with or without a bioabsorbable membrane in the tr	Amarante, ES; Leknes, KN; Skavland, J; Lie, T	JOURNAL OF PERIODONTOLOGY	2000	71	6	989	998	10.1902/jop.2000.71.6.989	63	2,52
63	CONTROLLED CLINICAL-EVALUATION OF THE SUBPEDICLE CONNECTIVE-TISSUE GRAFT	BORGHETTI, A; LOUISE, F	JOURNAL OF PERIODONTOLOGY	1994	65	12	1107	1112	10.1902/jop.1994.65.12.1107	63	2,03
64	Acellular dermal matrix allograft versus subepithelial connective tissue graft in treatment c	Moslemi, Neda; Jazi, Mahvash Mousavi; Haghig	JOURNAL OF CLINICAL PERIODONTOLOGY	2011	38	12	1122	1129	10.1111/j.1600-051X.2011.01789.x	62	4,43
65	A multicenter comparative study of two root coverage procedures: Coronally advanced fla	Nemcovsky, CE; Artzi, Z; Tal, H; Kozlovsky, A; Mc	JOURNAL OF PERIODONTOLOGY	2004	75	4	600	607	10.1902/jop.2004.75.4.600	62	2,95
66	Clinical evaluation of tetracycline HCl conditioning in the treatment of gingival recessions	Bouchard, P; Nilveus, R; Etienne, D	JOURNAL OF PERIODONTOLOGY	1997	68	3	262	269	10.1902/jop.1997.68.3.262	62	2,21
67	Coronally advanced flap plus connective tissue graft techniques for the treatment of deep	Zucchelli, Giovanni; Marzadori, Matteo; Mouns	JOURNAL OF CLINICAL PERIODONTOLOGY	2014	41	8	806	813	10.1111/jcpe.12269	61	5,55
68	Treatment of gingival recessions by combined periodontal regenerative technique, guided	Paolantonio, M	JOURNAL OF PERIODONTOLOGY	2002	73	1	53	62	10.1902/jop.2002.73.1.53	61	2,65
69	Coronally positioned flap for treatment of restored root surfaces: A 6-month clinical evalu	Lucchesi, Juliana Antico; Santos, Vanessa Ren	JOURNAL OF PERIODONTOLOGY	2007	78	4	615	623	10.1902/jop.2007.060380	60	3,33
70	Comparative 6-month clinical study of a semilunar coronally positioned flap and subepith	Bittencourt, Sandro; Ribeiro, Rica Del Peloso; S	JOURNAL OF PERIODONTOLOGY	2006	77	2	174	181	10.1902/jop.2006.050114	60	3,16
71	Evaluation of Human Recession Defects Treated With Coronally Advanced Flaps and Eithe	McGuire, Michael K.; Scheyer, E. Todd; Nunn, M	JOURNAL OF PERIODONTOLOGY	2012	83	11	1353	1362	10.1902/jop.2012.110373	59	4,54
72	Predictor factors for long-term outcomes stability of coronally advanced flap with or with	Rasperini, Giulio; Acunzo, Raffaele; Pellegrini, (	JOURNAL OF CLINICAL PERIODONTOLOGY	2018	45	9	1107	1117	10.1111/jcpe.12932	57	8,14
73	Periodontal Conditions of Sites Treated With Gingival Augmentation Surgery Compared W	Agudio, Giancarlo; Cortellini, Pierpaolo; Buti, J	JOURNAL OF PERIODONTOLOGY	2016	87	12	1371	1378	10.1902/jop.2016.160284	57	6,33
74	Clinical evaluation of coronally advanced flap with or without acellular dermal matrix graft	Ahmedbeyli, Cavid; Ipci, Sebnem Dirikan; Caka	JOURNAL OF CLINICAL PERIODONTOLOGY	2014	41	3	303	310	10.1111/jcpe.12211	57	5,18
75	Smoking may affect root coverage outcome: A prospective clinical study in humans	Martins, AG; Andia, DC; Sallum, AW; Sallum, EA	JOURNAL OF PERIODONTOLOGY	2004	75	4	586	591	10.1902/jop.2004.75.4.586	57	2,71
76	Fourteen-year outcomes of coronally advanced flap for root coverage: follow-up from a ra	Prato, Giovanpaolo Pini; Rotundo, Roberto; Fra	JOURNAL OF CLINICAL PERIODONTOLOGY	2011	38	8	715	720	10.1111/j.1600-051X.2011.01744.x	56	4
77	Root coverage in isolated gingival recessions using autograft versus allograft: A pilot study	Joly, Julio C.; Carvalho, Alexandre M.; da Silva, R	JOURNAL OF PERIODONTOLOGY	2007	78	6	1017	1022	10.1902/jop.2007.060428	56	3,11
78	Root coverage employing an envelope technique or guided tissue regeneration with a bioa	Müller, HP; Stahl, M; Eger, T	JOURNAL OF PERIODONTOLOGY	1999	70	7	743	751	10.1902/jop.1999.70.7.743	56	2,15
79	Clinical evaluation of Miller class I and II recessions treatment with the use of modified co	Pietruska, Malgorzata; Skurska, Anna; Podlews	JOURNAL OF CLINICAL PERIODONTOLOGY	2019	46	1	86	95	10.1111/jcpe.13031	55	9,17
80	Coronally positioned flap procedures with or without a biodegradable membrane in the tr	Leknes, KN; Amarante, ES; Price, DE; Boe, OE; S	JOURNAL OF CLINICAL PERIODONTOLOGY	2005	32	5	518	529	10.1111/j.1600-051X.2005.00706.x	55	2,75
81	A comparative study of coronally advanced flaps with and without the addition of enamel	Cueva, MA; Boltchi, FE; Hallmon, WW; Nunn, M	JOURNAL OF PERIODONTOLOGY	2004	75	7	949	956	10.1902/jop.2004.75.7.949	55	2,62
82	Gingival recession treatment with connective tissue grafts in smokers and non-smokers	Erley, Kenneth J.; Swiec, Gary D.; Herold, Rober	JOURNAL OF PERIODONTOLOGY	2006	77	7	1148	1155	10.1902/jop.2006.050252	53	2,79
83	Evaluation of the safety and efficacy of periodontal applications of a living tissue-enginee	Wilson, TG; McGuire, MK; Nunn, ME	JOURNAL OF PERIODONTOLOGY	2005	76	6	881	889	10.1902/jop.2005.76.6.881	53	2,65
84	EFFECTS OF TETRACYCLINE HCL CONDITIONING AND FIBRIN-FIBRONECTIN SYSTEM APPI	TROMBELLI, L; SCHINCAGLIA, GP; ZANGARI, F;	JOURNAL OF PERIODONTOLOGY	1995	66	5	313	320	10.1902/jop.1995.66.5.313	53	1,77
85	Comparison of two surgical procedures for use of the acellular dermal matrix graft in the t	Felipe, Maria Emilia M. C.; Andrade, Patricia F.; (	JOURNAL OF PERIODONTOLOGY	2007	78	7	1209	1217	10.1902/jop.2007.060356	52	2,89
86	Patient-reported outcomes and aesthetic evaluation of root coverage procedures: a 12-m	Stefanini, Martina; Jepsen, Karin; de Sanctis, M	JOURNAL OF CLINICAL PERIODONTOLOGY	2016	43	12	1132	1141	10.1111/jcpe.12626	51	5,67
87	Cervical restoration and the amount of soft tissue coverage achieved by coronally advance	Santamaria, Mauro Pedrine; Feitosa, Daniela da	JOURNAL OF CLINICAL PERIODONTOLOGY	2009	36	5	434	441	10.1111/j.1600-051X.2009.01389.x	51	3,19
88	Coronally positioned flap with or without acellular dermal matrix graft in the treatment of	Côrtes, AD; Martins, AG; Nociti, FH; Sallum, AW	JOURNAL OF PERIODONTOLOGY	2004	75	8	1137	1144		51	2,43
89	Citric acid demineralization and subepithelial connective tissue grafts	Caffesse, RG; De LaRosa, M; Garza, M; Munne-T	JOURNAL OF PERIODONTOLOGY	2000	71	4	568	572	10.1902/jop.2000.71.4.568	50	2
90	A 6-month comparative clinical study of a conventional and a new surgical approach for ro	Barros, RRM; Novaes, AB; Grisi, MFM; Souza, SL	JOURNAL OF PERIODONTOLOGY	2004	75	10	1350	1356	10.1902/jop.2004.75.10.1350	49	2,33
91	Acellular Dermal Matrix Seeded With Autologous Gingival Fibroblasts for the Treatment of	Jhaveri, Hirai M.; Chavan, Mahesh S.; Tomar, Ge	JOURNAL OF PERIODONTOLOGY	2010	81	4	616	625	10.1902/jop.2009.090530	48	3,2
92	Long-Term Results Comparing Xenogeneic Collagen Matrix and Autogenous Connective T	McGuire, Michael K.; Scheyer, E. Todd	JOURNAL OF PERIODONTOLOGY	2016	87	3	221	227	10.1902/jop.2015.150386	47	5,22
93	Implant Associated Soft Tissue Defects in the Anterior Maxilla: A Randomized Control Trial	Anderson, Lauren E.; Inglehart, Marita R.; El-Kh	IMPLANT DENTISTRY	2014	23	4	416	425	10.1097/ID.0000000000000122	47	4,27
94	Subepithelial Connective Tissue Graft for Treatment of Gingival Recessions With and With	Rasperini, Giulio; Roccuzzo, Mario; Francetti, L	INTERNATIONAL JOURNAL OF PERIODONTICS	2011	31	2	133	139		47	3,36
95	Treatment of gingival recession using a collagen membrane with or without the use of den	Kimble, KM; Eber, RM; Soehren, S; Shyr, Y; Wan	JOURNAL OF PERIODONTOLOGY	2004	75	2	210	220	10.1902/jop.2004.75.2.210	47	2,24
96	A clinical/statistical comparison between the subpedicle connective tissue graft method	Ricci, G; Silvestri, M; Tinti, C; Rasperini, G	INTERNATIONAL JOURNAL OF PERIODONTICS	1996	16	6	539	545		47	1,62
97	Long term assessment of root coverage stability using connective tissue graft with or with	Barootchi, Shayan; Tavelli, Lorenzo; Di Gianfilip	JOURNAL OF CLINICAL PERIODONTOLOGY	2019	46	11	1124	1133	10.1111/jcpe.13187	46	7,67
98	Connective tissue graft and tunnel or trapezoidal flap for the treatment of single maxillary	Santamaria, Mauro Pedrine; da Silva Neves, Fel	JOURNAL OF CLINICAL PERIODONTOLOGY	2017	44	5	540	547	10.1111/jcpe.12714	45	5,63
99	Concentrated growth factor in the treatment of adjacent multiple gingival recessions: a sp	Dogan, Seyma Bozkurt; Dede, Figen Ongoz; Bal	JOURNAL OF CLINICAL PERIODONTOLOGY	2015	42	9	868	875	10.1111/jcpe.12444	45	4,5
100	Treatment of gingival recession:: Comparison of two techniques of subepithelial connect	Tözüm, TF; Keçeli, HG; Güncü, GN; Hatipoglu, H	JOURNAL OF PERIODONTOLOGY	2005	76	11	1842	1848	10.1902/jop.2005.76.11.1842	45	2,25
101	Root coverage with connective tissue graft associated with coronally advanced flap or tun	Azaripour, Adriano; Kissinger, Maren; Farina, Vi	JOURNAL OF CLINICAL PERIODONTOLOGY	2016	43	12	1142	1150	10.1111/jcpe.12627	44	4,89
